# Supplementary material for: Multi‐dataset identification of innovative feature genes and molecular mechanisms in keratoconus
Source: J Cell Mol Med. 2024 Sep 19;28(18):e70079. doi: 10.1111/jcmm.70079 (PMC11412914; doi:10.1111/jcmm.70079)
Supplement: Supplementary file 2 — Material S2. [file JCMM-28-e70079-s002.zip › 7.GSEA analysis/2.ATOH7/KEGG_COLORECTAL_CANCER.html]

Details for gene set KEGG\_COLORECTAL\_CANCER[GSEA]

|  || Dataset | ATOH7.group.cls#h\_versus\_l |
| Phenotype | group.cls#h\_versus\_l |
| Upregulated in class | h |
| GeneSet | KEGG\_COLORECTAL\_CANCER |
| Enrichment Score (ES) | 0.3523576 |
| Normalized Enrichment Score (NES) | 1.3863817 |
| Nominal p-value | 0.10056926 |
| FDR q-value | 0.33188176 |
| FWER p-Value | 0.914 |
Table: GSEA Results Summary

  

Fig 1: Enrichment plot: KEGG\_COLORECTAL\_CANCER      
 Profile of the Running ES Score & Positions of GeneSet Members on the Rank Ordered List

  

| SYMBOL | TITLE | RANK IN GENE LIST | RANK METRIC SCORE | RUNNING ES | CORE ENRICHMENT || 1 | AKT3 | na | 9 | 1.043 | 0.1455 | Yes |
| 2 | AXIN2 | na | 285 | 0.630 | 0.2256 | Yes |
| 3 | LEF1 | na | 735 | 0.492 | 0.2814 | Yes |
| 4 | BCL2 | na | 1098 | 0.432 | 0.3313 | Yes |
| 5 | TGFB1 | na | 3047 | 0.278 | 0.3140 | Yes |
| 6 | TCF7 | na | 3059 | 0.277 | 0.3524 | Yes |
| 7 | MAP2K1 | na | 4240 | 0.228 | 0.3501 | No |
| 8 | APC | na | 5634 | 0.182 | 0.3353 | No |
| 9 | PIK3R3 | na | 6166 | 0.166 | 0.3431 | No |
| 10 | TCF7L1 | na | 6644 | 0.153 | 0.3507 | No |
| 11 | TGFB3 | na | 8550 | 0.107 | 0.3108 | No |
| 12 | DCC | na | 8701 | 0.104 | 0.3210 | No |
| 13 | ARAF | na | 10150 | 0.079 | 0.2903 | No |
| 14 | APC2 | na | 10749 | 0.070 | 0.2828 | No |
| 15 | MLH1 | na | 11553 | 0.058 | 0.2678 | No |
| 16 | TGFBR1 | na | 11693 | 0.057 | 0.2716 | No |
| 17 | MSH6 | na | 11757 | 0.056 | 0.2776 | No |
| 18 | RAC2 | na | 13916 | 0.031 | 0.2197 | No |
| 19 | BIRC5 | na | 14003 | 0.031 | 0.2215 | No |
| 20 | CASP9 | na | 14196 | 0.029 | 0.2200 | No |
| 21 | MSH2 | na | 14476 | 0.026 | 0.2156 | No |
| 22 | MAPK1 | na | 14536 | 0.025 | 0.2174 | No |
| 23 | TP53 | na | 14698 | 0.024 | 0.2161 | No |
| 24 | RAF1 | na | 15219 | 0.019 | 0.2038 | No |
| 25 | RHOA | na | 15382 | 0.018 | 0.2016 | No |
| 26 | AXIN1 | na | 15654 | 0.015 | 0.1959 | No |
| 27 | AKT1 | na | 15787 | 0.014 | 0.1941 | No |
| 28 | GSK3B | na | 16028 | 0.013 | 0.1890 | No |
| 29 | PIK3CD | na | 16202 | 0.011 | 0.1856 | No |
| 30 | CYCS | na | 16260 | 0.011 | 0.1854 | No |
| 31 | RAC1 | na | 16331 | 0.010 | 0.1848 | No |
| 32 | JUN | na | 16587 | 0.008 | 0.1786 | No |
| 33 | MAPK3 | na | 16821 | 0.006 | 0.1727 | No |
| 34 | MAPK8 | na | 17140 | 0.003 | 0.1640 | No |
| 35 | BAD | na | 17218 | 0.002 | 0.1621 | No |
| 36 | BAX | na | 17508 | 0.000 | 0.1538 | No |
| 37 | SMAD3 | na | 18063 | -0.005 | 0.1384 | No |
| 38 | CTNNB1 | na | 18214 | -0.006 | 0.1349 | No |
| 39 | MYC | na | 19651 | -0.019 | 0.0961 | No |
| 40 | MSH3 | na | 20028 | -0.022 | 0.0883 | No |
| 41 | TGFBR2 | na | 20164 | -0.023 | 0.0877 | No |
| 42 | CCND1 | na | 20546 | -0.027 | 0.0805 | No |
| 43 | PIK3CB | na | 20685 | -0.028 | 0.0805 | No |
| 44 | MAPK10 | na | 20989 | -0.032 | 0.0762 | No |
| 45 | PIK3CA | na | 21359 | -0.036 | 0.0705 | No |
| 46 | APPL1 | na | 21460 | -0.037 | 0.0727 | No |
| 47 | SMAD4 | na | 22118 | -0.044 | 0.0600 | No |
| 48 | TCF7L2 | na | 22134 | -0.044 | 0.0657 | No |
| 49 | PIK3R2 | na | 22209 | -0.045 | 0.0699 | No |
| 50 | MAPK9 | na | 24826 | -0.082 | 0.0060 | No |
| 51 | CASP3 | na | 24828 | -0.083 | 0.0175 | No |
| 52 | TGFB2 | na | 24944 | -0.084 | 0.0259 | No |
| 53 | BRAF | na | 25048 | -0.086 | 0.0350 | No |
| 54 | KRAS | na | 25083 | -0.087 | 0.0461 | No |
| 55 | SMAD2 | na | 25145 | -0.088 | 0.0566 | No |
| 56 | PIK3CG | na | 26210 | -0.109 | 0.0411 | No |
| 57 | RALGDS | na | 26371 | -0.112 | 0.0522 | No |
| 58 | AKT2 | na | 27111 | -0.128 | 0.0488 | No |
| 59 | PIK3R1 | na | 27720 | -0.144 | 0.0513 | No |
| 60 | FOS | na | 27908 | -0.149 | 0.0666 | No |
| 61 | RAC3 | na | 32406 | -0.320 | -0.0184 | No |
| 62 | PIK3R5 | na | 34383 | -0.611 | 0.0100 | No |
Table: GSEA details [plain text format]

  

Fig 2: KEGG\_COLORECTAL\_CANCER      
 Blue-Pink O' Gram in the Space of the Analyzed GeneSet

  

Fig 3: KEGG\_COLORECTAL\_CANCER: Random ES distribution      
 Gene set null distribution of ES for **KEGG\_COLORECTAL\_CANCER**
